# Sacroiliac joint variation associated with diffuse idiopathic skeletal hyperostosis

**DOI:** 10.1186/s12891-020-3105-z

**Published:** 2020-02-10

**Authors:** Yasuhito Yahara, Taketoshi Yasuda, Yoshiharu Kawaguchi, Kayo Suzuki, Shoji Seki, Miho Kondo, Hiroto Makino, Katsuhiko Kamei, Masahiko Kanamori, Tomoatsu Kimura

**Affiliations:** 1 Department of Orthopaedic Surgery, Faculty of Medicine, University of Toyama, 2630 Sugitani, Toyama, 930-0194 Japan; 2 Department of Human Science 1, University of Toyama, 2630 Sugitani, Toyama, 930-0194 Japan

**Keywords:** Diffuse idiopathic skeletal hyperostosis, Sacroiliac joint, Degenerative lumbar disease, Anterior paraarticular bridging

## Abstract

**Background:**

Diffuse idiopathic skeletal hyperostosis (DISH) is characterized by the ossification of vertebral bodies and peripheral entheses. However, variations in sacroiliac (SI) joint change in patients with DISH have not been fully clarified. The purpose of this study was to evaluate SI joint variation in patients with DISH in comparison with a non-DISH population.

**Methods:**

A total of 342 SI joints in 171 patients (DISH+, *n* = 86; DISH-, *n* = 85) who had undergone lumbar spine surgery were analyzed by computed tomography examination. SI joint variations were classified into four types: Type 1, normal or tiny peripheral bone irregularity; Type 2, subchondral bone sclerosis and osteophytes formation; Type 3, vacuum phenomenon; and Type 4, bridging osteophyte and bony fusion. The type of bridging osteophyte in SI joints and the prevalence of ossification in each spinal segment from C1 to SI joint were also examined.

**Results:**

The most common SI joint variation in the DISH+ group was bony fusion (Type 4), with 71.6% exhibiting anterior paraarticular bridging. On the other hand, SI joint vacuum phenomenon (Type 3) was the most frequent change (57.1%) in the DISH- group. The middle to lower thoracic spine and SI joints were highly affected in DISH and caused bony ankylosis.

**Conclusions:**

Anterior paraarticular bridging was the most common type of SI joint change in patients with DISH who underwent lumbar spine surgery. The present results regarding variations of SI joint changes in DISH should help understand the etiology of DISH.

## Background

Diffuse idiopathic skeletal hyperostosis (DISH) is a skeletal disorder characterized by ossification and calcification along the anterolateral aspect of vertebral bodies and peripheral entheses [1–3]. In 1976, Resnick et al. proposed diagnostic criteria for DISH in the spine based on radiographic features, requiring: 1) ossification of at least four contiguous vertebral bodies; 2) relative preservation of the intervertebral disc space; and 3) absence of apophyseal joint bony ankylosis and sacroiliac (SI) joint erosion, sclerosis, or intraarticular osseous fusion [3]. Those hallmarks are not limited to the spine, and extraspinal manifestations have been reported, such as hyperostosis at the rotator cuff, deltoid tuberosity of the humerus, hand, ulnar olecranon, pelvis, and patella [4–7].

DISH also affects the SI joints [2, 3, 8, 9]. The SI joints connect the sacrum and ilium, and play an essential role in strong weight-bearing and effective load transfer between the spine and legs [10]. Stabilities of the SI joints are maintained mainly through a combination of bone structures and strong intrinsic and extrinsic ligaments. Those ligaments enclose the cartilaginous parts of the SI joints and therefore represent a major site of entheses [11]. Although SI joint involvement in DISH is characterized by radiographic osteophytes, paraarticular bony bridging and coexistent osteoarthritis [2, 9], these conditions should be distinguished from sacroiliitis due to ankylosing spondylitis (AS).

AS is a type of arthritis affecting the spine and SI joint in the relatively young adult population [12]. Although both DISH and AS share the features of bone proliferation and ankylosis in the spine and peripheral entheses, the hallmarks of bone proliferation of SI joint are dissimilar. Radiographic sacroiliitis is the indispensable feature of the modified New York criteria for the diagnosis of AS [13]. Sacroiliitis in AS is characterized by SI joint erosion, sclerosis, and “intra”-articular osseous fusion, represented as the negative feature of DISH in the original Resnick criteria [9]. In contrast, “para”-articular bony fusion and osteophyte formation in SI joint are frequently observed in DISH. Although some authors have noted the differences in SI joint involvement between those two entities [9, 12, 14], the low awareness of SI joint variations still leads to confusion regarding SI joint changes in DISH and misunderstanding that SI joint involvement is absent or SI joints are normal in DISH.

The aims of the present study were to evaluate SI joint variation in patients with DISH (DISH+ group) or without DISH (DISH- group) who underwent lumbar spine surgery and to clarify differences in SI joint variation between DISH+ and DISH- groups.

## Methods

**Fig. 1:**
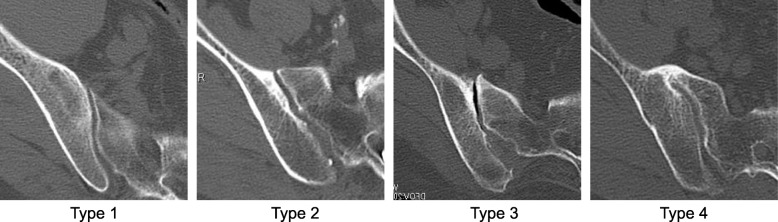
Variation of sacroiliac joint changes on computed tomography. Type 1, normal or tiny peripheral bone irregularity; Type 2, subchondral bone sclerosis and osteophytes formation; Type 3, vacuum phenomenon of SI joint; Type 4, bridging osteophyte and bony fusion of SI joint

**Fig. 2:**
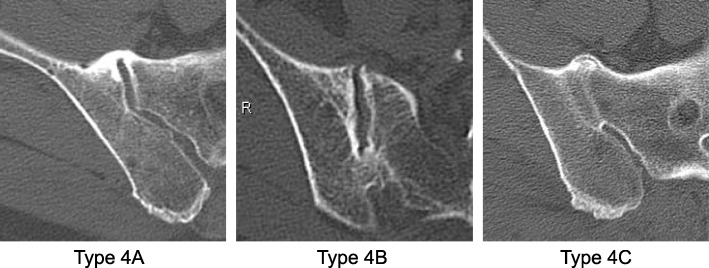
Sub-classification of sacroiliac joint ankylosis

For the present study, we retrospectively reviewed 504 patients who had undergone lumbar spine surgery between 2009 and 2016 in our hospital. Radiograms and computed tomography (CT) of the total spine were performed before surgery. Reconstructed sagittal and axial views of the total spine and cranial part of the SI joint were evaluated. Patients diagnosed with spinal tumor, trauma, autoimmune disease, or pyogenic discitis were excluded. Further, patients < 52 years old were excluded from the study to eliminate the AS population. In this study, we defined DISH as the radiographic and CT finding of ossification along the anterolateral aspects of at least 4 contiguous levels with relative presentation of disc height. Patients diagnosed with DISH according to our criteria were allocated to the DISH+ group. Age- and sex-matched control patients without DISH were included in the DISH- group. According to CT findings, SI joint variations were divided into 4 types: Type 1, normal or tiny peripheral bone irregularity; Type 2, subchondral bone sclerosis and osteophyte formation; Type 3, vacuum phenomenon of SI joint; and Type 4, bridging osteophyte and bony fusion of SI joint (Fig. 1). We also further classified Type 4 into three subgroups depending on the site of bony ankylosis, as previously described [15]: anterior paraarticular bridging (Type 4A), posterior paraarticular bridging (Type 4B), and intraarticular ankylosis (Type 4C) (Fig. 2). CT images of the SI joint were evaluated by two orthopaedic surgeons. To calculate interobserver error (Fleiss’ k score) and intraobserver error (Cohen’s k score), three blinded orthopaedic surgeons evaluated CT images of the SI joint. Based on the range of ossification sites, DISH was also classified into 5 types as a modification of a previously reported system: cervical, thoracic, thoracolumbar, lumbar, or diffuse type [16]. Cervical, thoracic, and lumbar types indicated that ossification along more than 4 contiguous vertebral bodies existed only within C1-C7, T1-T12, or L1-L5, respectively. Thoracolumbar type was defined in patients showing ossification along more than 4 vertebral bodies within the T1-L5 level. Diffuse type indicated ossification more than 4 contiguous vertebral bodies within the C1-L5 level. The prevalence of ossification in each spinal segment from C1 to the SI joint and the lower vertebral end of ossification were determined from sagittal and axial images using reconstructed CT. In this analysis, SI joint ossification represented either uni- or bilateral SI joint bony fusions. This study was approved by the ethics committee at Toyama University Hospital.

Data are shown as mean and standard deviation. Significant differences between means were analyzed using Student’s t-test (two-sided) and the chi-square test, as appropriate. Statistical analysis was performed using Excel statistical software (Statcel3; OMS, Tokorozawa, Japan). Values of *P* < 0.05 were considered statistically significant. To calculate the interobserver error (Fleiss’ k score) and intraobserver error (Cohen’s k score), R version 3.5.3 and package irr (version 0.84.1) were used.

## Results

The characteristics of patients are shown in Table 1. Eighty-six of the 504 patients (17.0%) were diagnosed with DISH according to our criteria. Variations in bilateral SI joints in 86 DISH+ patients [68 males, 18 females; mean age, 72.9 ± 7.1 years, total 172 SI joints] and 85 age- and sex-matched DISH- patients [65 males, 20 females; mean age, 72.6 ± 7.5 years, total 170 SI joints] were evaluated. The prevalence of DISH was significantly higher in males (79.1%) than in females (20.9%). In terms of clinical manifestations and operative procedures, no significant differences were identified between DISH- and DISH+ groups.

**Table 1:** Characteristic of patients

	DISH-	DISH+	p
Number of patients	85	86	–
Male (%) / Female (%)	65 (76.4%) / 20 (23.6%)	68 (79.1%) / 18 (20.9%)	0.44
Mean age	72.6 ± 7.5	72.9 ± 7.1	0.41
Male / Female	72.0 ± 7.9 / 74.8 ± 5.5	72.5 ± 7.4 / 74.5 ± 6.1	–
Lumbar spine disease			
Spinal stenosis (%)	71 (83.5%)	73 (84.9%)	0.22
Spondylosis (%)	4 (4.7%)	8 (9.3%)	
Disc herniation (%)	10 (11.8%)	5 (5.8%)	
Operation			
Decompression / Fixation	44 / 41	44 /42	0.93

*DISH* diffuse idiopathic skeletal hyperostosis

**Fig. 3:**
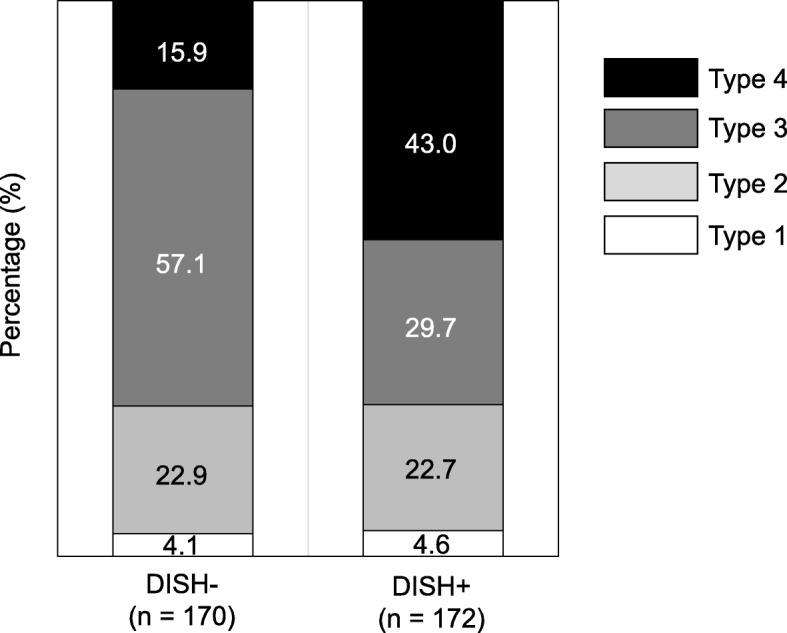
Prevalence of sacroiliac joint variation in patients with diffuse idiopathic skeletal hyperostosis and control subjects

The prevalence of SI joint variation is shown in Fig. 3. Bridging osteophyte and ankylosis (Type 4) was observed in 43.0% of SI joints in the DIHS+ group. Conversely, those changes were uncommon (15.9%) in the DISH- group. The SI joint vacuum phenomenon (Type 3) was the most frequent change in the DISH- group (57.1%). Anterior paraarticular bridging (Type 4A) was identified in 71.6% of ankylotic SI joints in the DISH+ group and 81.5% in the DISH- group (Table 2). Interobserver error was 0.751 and intraobserver error was 0.813.

**Table 2:** Sub-classification of Type 4 sacroiliac joint ankylosis

	DISH-	DISH+
Number of SI joints	27	74
Anterior (Type 4A, %)	22 (81.5%)	53 (71.6%)
Posterior (Type 4B, %)	0 (0%)	4 (5.4%)
Intraarticular (Type 4C, %)	5 (18.5%)	17 (23.0%)

*DISH* diffuse idiopathic skeletal hyperostosis; *SI* sacroiliac

**Fig. 4:**
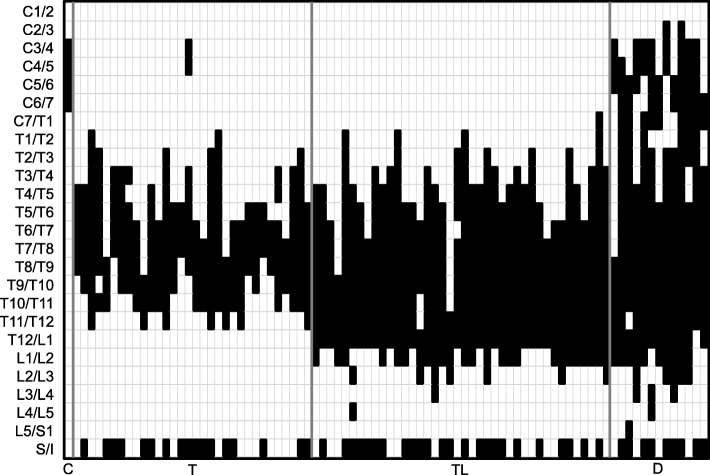
Ossification level of individual patients with diffuse idiopathic skeletal hyperostosis. C, cervical type; T, thoracic type; TL, thoracolumbar type; D, diffuse type

**Fig. 5:**
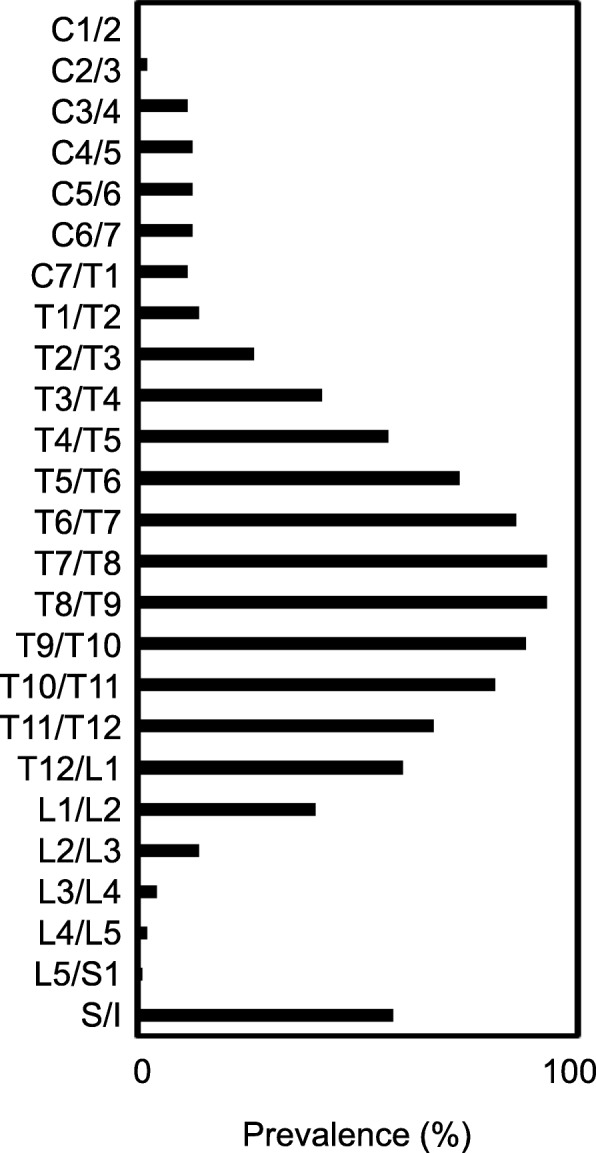
Prevalence of vertebral ossification in each spinal segment

**Fig. 6:**
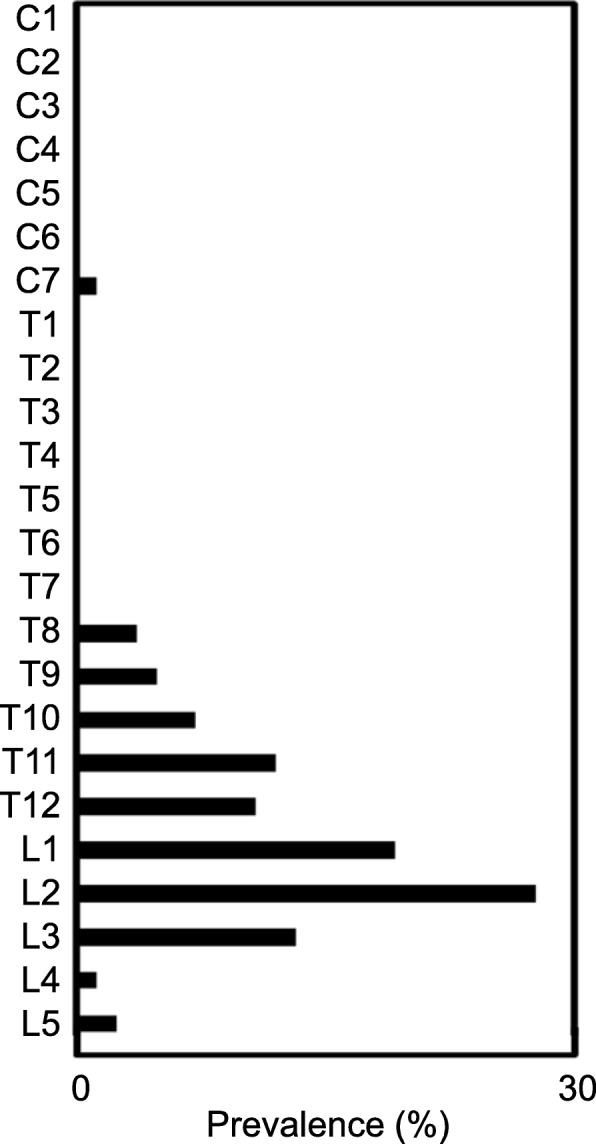
Level of the lower end of vertebral ossification

Ossification was seen along the thoracolumbar level in 46.5% of DISH patients, and along the thoracic level in 37.2% (Table 3). Thoracic and thoracolumbar levels can thus represent a major site of ankylosis in patients with DISH. In terms of sex, males showed a higher tendency toward diffuse-type ossification (17.6%) compared with females (5.5%). To clarify the relationship between the level of spinal ankylosis and SI joint change, we examined the distribution of ossification in each vertebral segment from C1 to the SI joint in individual patients (Fig. 4). The middle to lower thoracic spine (T5-L1) was more affected than other levels and showed ossification due to DISH. More importantly, the SI joint also tended to show a high rate of bony bridging and ossification, independent of the tendencies of another spinal segment (Fig. 5). Further, our data revealed that the lower end of vertebral ossification ranged from the thoracolumbar junction to the upper lumbar spine, with L2 (26.9%) as the most frequent terminal site of ossification (Fig. 6).

**Table 3:** Type of diffuse idiopathic skeletal hyperostosis classified by site of ossification

	Male	Female	Total
Number of patients	68	18	86
Cervical type (%)	0	1 (5.5%)	1 (1.2%)
Thoracic type (%)	23 (33.8%)	9 (50.0%)	32 (37.2%)
Thoracolumbar type (%)	33 (48.5%)	7 (38.9%)	40 (46.5%)
Lumbar type (%)	0	0	0
Diffuse type (%)	12 (17.6%)	1 (5.5%)	13 (15.1%)

## Discussion

This study analyzed the prevalence of SI joint variations in DISH+ and DISH- patients who had undergone lumbar spine surgery. We demonstrated that bony bridging and ankylosis of the SI joint were frequently observed in DISH+ patients compared with DISH- patients. Further, anterior paraarticular bridging of the SI joint was the most common type of SI joint change. The middle to lower thoracic spine and SI joint were highly affected by DISH and introduced bony ankylosis. In addition, the lower end of vertebral ossification of DISH terminated from the thoracolumbar junction to the upper lumbar spine.

Stability of the SI joint is maintained through a combination of only some bony structures and very strong intrinsic and extrinsic ligaments [10]. The proximal and ventral aspects of the SI joints are connected with the ventral sacroiliac ligament (VSIL) and proximal sacroiliac ligament (PSIL), representing synovial joints [11]. On the other hand, the superior and posterior aspects contained strong fibrous joint spaces with interosseous ligaments. These ligaments produce the multidirectional and structural stability of the SI joint. Both the VSIL and PSIL connect with the border of the iliac and sacral cartilage. The transition zone from ligament to cartilage comprises fibrocartilage representing entheses. Entheses contain fibroblasts, chondrocytes, collagen fibers, and calcified matrix. Entheses could thus represent a site of endochondral ossification, resulting in paraarticular bony bridging of SI joints [17].

Our study revealed that the spinal level from the middle to lower thoracic spine and SI joints were highly affected by DISH and introduced bony ankylosis. We also found that ossification of vertebrae due to DISH terminated from the thoracolumbar to upper lumbar segment. Such ossified segments could presumably act as long lever arms, increasing mechanical stress on the lower lumbar spine, following lumbar spinal degeneration and hypertrophy of the ligamentum flavum [18]. Non-fused lower lumbar segments could thus represent major sites of lumbar spinal stenosis and disc herniation associated with DISH. Kagotani et al. reported the presence of DISH as significantly associated with the presence of lumbar spondylosis [16]. Further, Yamada et al. demonstrated DISH as a risk factor for LSS requiring surgery [19]. Although the contribution of DISH to the severity of lumbar spinal disorders remains unclear, mechanical overloading below ankylosed sites may be a key contributor to lumbar spinal stenosis in patients with DISH.

In terms of surgical treatments for lumbar spinal disorders accompanying DISH, Otsuki et al. reported short-segment lumbar interbody fusion as a factor in delayed pseudarthrosis and adjacent segment disease (ASD) [20]. Further, numerous studies have reported that surgical treatment for traumatic spine fracture accompanying DISH often requires multi-level fusion to avoid postoperative ASD [21–23]. To maintain postoperative sagittal alignment, pelvic screw insertion, as a strong anchor of spinal fixation, became an indispensable technique not only in patients with DISH, but also in many clinical scenarios such as adult degenerative scoliosis, flat-back syndrome and kyphosis [24]. S2-Alar-Iliac (S2-AI) instrumentation has spread rapidly as a pelvic anchoring method for penetrating the SI joint. Compared to the iliac screw, the advantage of the S2-AI method includes a lower profiling setting of the screw, less extensive dissection of tissue, and higher pullout resistance [25, 26]. Elder et al. reported use of the S2-AI as an independent predictor of preventing reoperation and surgical site infection [27]. However, the long-term influence of SI joint fixation remains unclear. According to our recent data, DISH+ patients frequently exhibited SI joint ankylosis. S2-AI fixation, traversing and disrupting the SI joint, thus would not represent a disadvantage for DISH patients with SI joint ankylosis. Knowledge of the presence and variations of SI joint changes could be helpful for deciding on operative procedures.

AS, which belongs to a group of related diseases termed spondyloarthritides (SpA) [11, 17], is widely known to also affect the SI joint and introduces ankylosis. Although both DISH and AS share several clinical and radiographic features in the spine, the characteristics of bone proliferation differ [12, 14]. AS introduces ossification within the peripheral part of the annulus fibrosus in the intervertebral discs. On the other hand, ossification of the anterior longitudinal ligament and adjacent connective tissue is common in DISH, but not generally observed in AS. Typical findings of the SI joint in AS include sacroiliitis including joint erosions, joint space narrowing, sclerosis, and intraarticular ankylosis, but none of these are common in DISH [8, 12]. According to recent progress in the treatment of SpA using biological disease-modifying antirheumatic drugs, including tumor necrosis factor inhibitors (TNFi) and interleukin 17 inhibitors (IL-17i) [28, 29], early diagnosis facilitates timely treatment and may minimize structural damage. The present findings may thus contribute to an understanding of radiographic changes in the SI joint associated with DISH and sacroiliitis from SpA.

Some limitations of this study must be considered. First, the evaluation of cases was retrospective, and the populations of both groups were limited to patients who had undergone lumbar spine surgery. Analysis of a general population would also be worthwhile to confirm SI joint alterations due to DISH. Second, general health status and histories, such as obesity and diabetes mellitus, were not the focus of this study. Relationships between clinical symptoms and SI joint alterations therefore need to be elucidated in future studies. Third, criteria for diagnosing DISH from CT have not been established.

## Conclusions

In summary, we have presented SI joint variation due to DISH in patients who had undergone lumbar spine surgery. Anterior bony bridging and ankylosis of the SI joint are more frequent among patients with DISH. Further, the middle to lower thoracic spine and SI joint were highly affected by DISH, resulting in bony ankylosis. Clarification of the presence and variation of SI joint changes may lead to a better understanding of the etiology of DISH and improvements in clinical decision making.

## Data Availability

The datasets used and/or analyzed during the present study are available from the corresponding author on reasonable request.

## Abbreviations

AS: Ankylosing spondylitis
ASD: Adjacent segment disease
CT: Computed tomography
DISH: Diffuse idiopathic skeletal hyperostosis
IL-17i: Interleukin 17 inhibitors
PSIL: Proximal sacroiliac ligament
S2-AI: S2-Alar-Iliac
SI: Sacroiliac
SpA: Spondyloarthritides
TNFi: Tumor necrosis factor inhibitors
VSIL: Ventral sacroiliac ligament
